# Amelioration of Endotoxin-Induced Inflammatory Toxic Response by a Metal Chelator in Rat Eyes

**DOI:** 10.1167/iovs.17-22172

**Published:** 2018-01

**Authors:** Mohammad Shoeb, Min Zhang, Tianlin Xiao, Misha F. Syed, Naseem H. Ansari

**Affiliations:** 1 Departments of Biochemistry and Molecular Biology, University of Texas Medical Branch, Galveston, Texas, United States; 2 Ophthalmology and Visual Science, University of Texas Medical Branch, Galveston, Texas, United States

**Keywords:** inflammation, uveitis, metal chelation, EDTA, oxidative stress, eyes

## Abstract

**Purpose:**

Metal ions play a key role in exacerbating toxicity associated with oxidative stress and inflammation. This study examines the effects of a formulation containing the metal chelator ethylenediaminetetraacetic acid (EDTA) and permeability enhancer methyl sulfonyl methane (MSM) on the early course of inflammation in endotoxin-induced uveitis (EIU). The proprietary MSM/EDTA formulation of Livionex, Inc., which was used for this study, is covered by several patents and pending patent applications.

**Methods:**

EIU was induced by using subcutaneous injection of lipopolysaccharide (LPS) into the thighs of Lewis rats. Treatment consisted of topical application to the eyes of either PBS or eye drops designated as ME that contain EDTA and MSM. Clinical signs of uveitis were monitored at 6 and 24 hours postinjection. Oxidative and inflammatory markers were evaluated by ELISA or immunohistochemistry.

**Results:**

Rats treated with ME showed fewer clinical signs of uveitis including reduced miosis, fibrinous exudates, and dilated blood vessels. The aqueous humor of treated rats contained fewer leukocytes, lower protein levels, and less PGE_2_. Formation of protein adducts with the lipid peroxidation end-product, 4-hydroxynonenal, expression of NF-κB, TNF-α, and MMP-9 were all reduced in rats treated with ME.

**Conclusions:**

Our results indicate that ME eye drops downregulate the ocular inflammatory response in LPS treated rats, suggesting that induction of EIU involves metal ions and chelation therapy with ME is a potential treatment for uveitis.

Uveitis is an inflammation of any part of the uveal tract of the eye (iris, ciliary body and/or choroid). It is a common cause of vision loss, accounting for 10% to 15% of all cases of blindness worldwide and affects individuals regardless of age, sex, or race.^1^ Uveitis can result from complications of autoimmune diseases, bacterial infections, viral infections, and chemical and metabolic injuries associated with a variety of molecular and biochemical events that lead to ocular inflammation. Clinical cases have also been described in which no cause could be determined (idiopathic uveitis).^2^ Complications of uveitis that may lead to blindness include cataract, glaucoma, band keratopathy, vitreous opacities, retinal scars or detachment, vascular abnormalities, macular edema, and optic atrophy.^2^ Although the initial events leading to uveitis are not always clear, the eventual loss of vision has been ascribed to the ocular tissue damage caused by amplification of inflammatory processes.

Inflammation disturbs the pro-oxidant/antioxidant balance.^3^ Reactive oxygen species (ROS) formed during oxidative stress act as second messengers to stimulate numerous inflammatory pathways (Fig. 1). In one cascade, ROS catalyzed by metal ions, usually ferrous iron (Fe^2+^) induces lipid peroxidation in cell membranes. Production of one of the major toxic by-products of lipid peroxidation, 4-hydroxynonenal (HNE), is itself catalyzed by Fe^2+^.^4^ HNE subsequently conjugates to specific amino acids forming toxic protein-HNE adducts. In a second cascade associated with inflammation, release of intracellular Ca^2+^ stores by oxidative stress stimulates the activation of the transcription factor NF-κB.^5^ Ca^2+^ stimulates the degradation of IκB that allows the release of NF-κB to the nucleus where it activates gene transcription of a large number of cytokines, chemokines and other factors including cyclooxygenase-2 (COX-2) and TNF-α.^6,7^ COX-2 is the rate-limiting enzyme in the production of PGE_2_, a molecule involved in mediation of inflammation and thought to be essential for the breakdown of the blood-ocular barrier seen in uveitis.^8^ TNF-α is recognized as a central mediator in the pathophysiology of many chronic inflammatory diseases that cause increased risk of uveitis. TNF-α is a so-called “master cytokine” that controls many other cytokines,^9^ including forming a positive feedback loop to increase the activation of NF-κB. One important molecule involved in uveitis that is modulated by TNF-α is MMP-9. MMP-9 cleaves type IV collagen in basement membranes and allows the extravasion of leukocytes from the circulatory system into the aqueous humor during uveitis.^10^ Furthermore, various antioxidants and inhibitors of NF-κB that prevent expression of inflammatory cytokines have been shown to prevent uveitis and suggest the involvement of ROS in ocular inflammation.^11–15^

**Figure 1:**
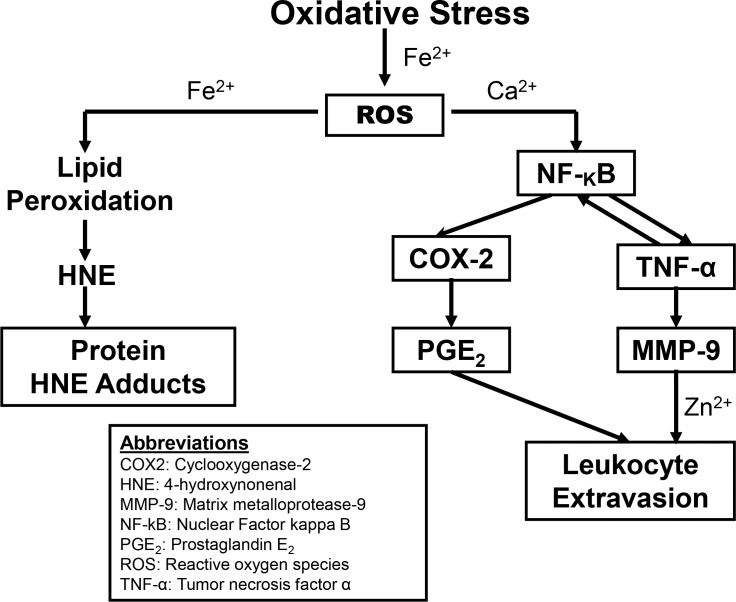
Involvement of divalent cations in inflammatory pathways important in uveitis. Oxidative stress caused by injection of LPS results in the metal ion catalyzed production of ROS. In the left-hand cascade, ROS catalyzed by ferrous ions, induces lipid peroxidation in cell membranes. One of the major toxic byproducts is HNE. HNE conjugates to specific amino acids forming toxic protein-HNE adducts. In the right-hand cascade, ROS stimulate the Ca^2+^ dependent release of NF-κB to the nucleus where it activates gene transcription of a large number of factors including COX-2 and TNF-α. COX-2 is the rate-limiting step in the production of PGE_2_. TNF-α also increases the activation of NF-κB. TNF-α modulates the production of MMP-9. Molecules in boxes were examined in this study.

Because of their direct participation in oxidative stress-linked disease processes, reduction or removal of transition metal and calcium ions potentially has anti-inflammatory effects.^16–18^ Removal of Fe^2+^ may block the Fenton reaction preventing formation of damaging •OH radicals. Removing Ca^2+^ may downregulate the production of calcium sensitive proteases that lead to NF-κB activation. Removing Zn^2+^ may reduce leukocyte extravasion. The purpose of this study is to determine the effects of the metal chelator EDTA in inhibiting inflammation in uveitis. LPS, a component of Gram-negative bacterial cell walls, induces acute ocular inflammation leading to uveitis in Lewis rats. This endotoxin-induced uveitis (EIU) is an animal model of acute uveitis in humans.^19^ Eye drops (ME) contained EDTA with a novel membrane permeation enhancer, MSM that allows localized penetration of EDTA into eye tissue.^20^ Topical application of ME to the rat eyes ameliorated diabetic cataract and associated oxidation-induced toxicity.^21^ Such a treatment was also able to attenuate neurodegenerative changes in the rat glaucomatous eye as evaluated by optic nerve and retinal morphology, and oxidative and inflammatory markers.^22^ In the present study, we have shown that topical treatment of EIU in Lewis rats with ME diminishes the inflammatory cell infiltration in aqueous humor, the production of inflammatory cytokines and oxidative stress as measured by protein-HNE immunostaining. The results suggest metal chelation therapy as a possible therapeutic intervention of ocular inflammation.

## Materials and Methods

### Chemicals and Reagents

PGE_2_ assay kits and protein-HNE antibodies were purchased from Cayman Chemical, Inc. (Ann Arbor, MI, USA). EDTA, MSM, and LPS derived from Escherichia coli were purchased from Sigma-Aldrich Corp. (St. Louis, MO, USA). Antibody against TNF-α was obtained from Calbiochem (San Diego, CA, USA) and antibodies to NF-κB and MMP-9 were obtained from Cell Signaling (Danvers, MA, USA). Biotinylated secondary antibody and DAB+ substrate chromogen were obtained from the LSAB+System-HRP kit (Dako North America, Inc., Carpinteria, CA, USA).

### Induction and Treatment of EIU in Rats

We obtained 6- to 8-week-old male Lewis rats weighing approximately 150 to 160 g from Harlan Laboratories (Houston, TX, USA) and randomly divided them into three groups (*n* = 6) for each of the time points. Animals were kept in the University of Texas Medical Branch Animal Research Center. All of the animal studies were conducted in compliance with the ARVO Statement for the Use of Animals in Ophthalmic and Vision Research. To induce EIU, Escherichia coli LPS (200 μg) dissolved in 100 μL PBS, pH 7.4, was subcutaneously injected at two locations on the rear thighs of the animals. Rats in the control group were injected with vehicle (PBS). In the preliminary experiments, eye drops containing 0.25% EDTA and 0.54% MSM, a formulation that ameliorated diabetic cataract when applied topically to the eye,^21^ did not display any therapeutic efficacy. In this study (acute), higher concentrations of EDTA (1.25%) and MSM (2.7%) were used and this formulation was designated as ME. Applications of ME eye drops were begun immediately after the LPS injection and were applied every 2.5 hours throughout the 6-hour experiments conducted to evaluate early inflammatory response and every 4 hours during the 24-hour experiments to evaluate oxidative and inflammatory parameters and clinical manifestation. PBS was applied to the eyes of the control rats at the same times. MSM alone did not display any therapeutic efficacy in the initial experiments (data provided in the Supplementary Material)

### Clinical Scoring of Inflammation in Rat Eyes

Clinical scoring of uveitis was conducted at the 24-hour timepoint and accomplished by anesthetizing the rat and examining the eyes under a dissecting microscope. The classical visible signs of ocular inflammation and severity were evaluated by an expert ophthalmologist who was blinded to the experimental groups and examined the eyes using slit lamp biomicroscopic device; pictures were taken with a digital camera (Sony Cyber-Shot 7.2 Mega Pixel; Sony Corp., Tokyo, Japan). Indeed, evaluation of uveitis clinical signs has been subjective in the preclinical studies; however, it was carefully and blindly conducted. Scoring for severity was done on a scale of 0 to 3. A score of 0 represents no contraction of the small pupil (SP), no dilation or redness of the iris blood vessels; and no deposition of the fibrinous membrane (FM) exudates. Scores of 3, 2, and 1: (SP) 100%, 50%, and 25% contraction of the pupil, respectively; (DBV) intense dilation of the main blood vessel and redness, dilation and redness 50% of the score 3, and redness but no dilation of the main vessels, respectively; (FM) Approximately 3%, 2%, and 1% area of the cornea, respectively. For confidence, scoring was also done by another evaluator in a blinded manner. The rats were then used for collection of aqueous humor and histology and immunohistochemistry of the eyes.

### Infiltrating Cells and Proteins in Aqueous Humor

The rats were euthanatized 6 and 24 hours after LPS injection, and aqueous humor was collected from the eyes immediately by anterior chamber puncture with a 30-gauge needle under a surgical microscope. The 24-hour samples were stored on ice until testing, and cell counts and total protein concentrations were measured on the day of sample collection. An aliquot was suspended in an equal amount of trypan-blue solution, and cells counted with a hemocytometer under a light microscope (Olympus Optical Ltd., London, UK). The total protein concentration in the aqueous humor samples was measured with a protein assay kit using the Bradford method (Bio-Rad, Hercules, CA, USA). The 6-hour aqueous humor was stored at –80°C. Level of PGE_2_ in the thawed aqueous humor was measured in duplicate using a commercially available enzyme ELISA kit (R&D Systems, Minneapolis, MN, USA) according to the manufacturer's instructions.

### Histology and Immunohistochemistry

The Paraffin-embedded sections were warmed at 60°C for 1 hour and deparaffinized in xylenes, rehydrated, and washed in deionized water. After endogenous peroxidase was blocked with 3% H_2_O_2_, the sections were rinsed in PBS twice and incubated with blocking buffer (2% BSA, 0.1% Triton X-100, 2% normal rabbit IgG, and 2% normal goat serum) overnight at 4°C. Some sections were stained with hematoxylin and eosin (H&E) to visualize morphology and leukocytes. Other sections were incubated with antibodies against protein-HNE (dilution 1:1000); TNF-α (dilution 1:200); and MMP-9 (dilution 1:100). The 6-hour sections were incubated with antibodies against NF-kB (dilution 1:100). Sections were incubated in HRP-conjugated secondary antibody and the peroxidase reaction was visualized using diaminobenzidine (DAB).

### Statistical Analysis

Data are expressed as the mean ± SEM. Groups were compared with ANOVA using the Bonferroni post hoc test for significance between groups using statistical software (GraphPad Prism; GraphPad Software, Inc., La Jolla CA, USA) A value of *P* < 0.05 was considered statistically significant.

## Results

### Inflammation, Leukocyte Infiltration and Protein Concentration in Aqueous Humor

**Figure 2:**
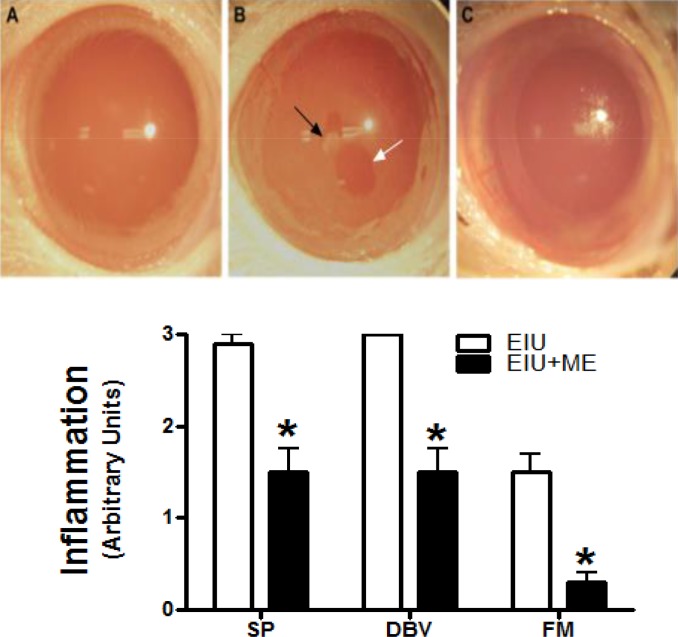
Reduction of miosis, membrane exudates and dilation of blood vessels by ME. The severity of EIU and inflammation was scored on a scale of 0 to 3 by slit lamp biomicroscopic examination by an expert ophthalmologist who was blinded to the experimental groups. (A) Rat eye pictures were taken 24 hours after LPS injection. (A) Control. (B) EIU. (C) EIU + ME. Black arrow, fibrinous exudate, white arrow, small pupil. (b) Clinical inflammation scores after 24 hours of EIU. Scoring for severity was done on a scale of 0 to 3. Score of 0 represents no contraction of the SP; no DBV; and no deposition of the FM exudates. Score of 3, 2, and 1: (SP) 100%, 50%, and 25% contraction of the pupil, respectively; (DBV) intense dilation of the main blood vessel and redness, dilation and redness 50% of the score 3, and redness but no dilation of the main vessels, respectively; (FM) Approximately 3%, 2%, and 1% area of the cornea, respectively. For confidence, scoring was also done by another evaluator in a blinded manner. *Significantly different from EIU, P < 0.05.

We examined rat eyes 24 hours after LPS inoculation for clinical symptoms of uveitis. The clinical symptoms were scored for miosis or SP, increased formation of dilated blood vessels (DBV); and FM exudates on a scale of 0 to 3 where 0 represents no SP, DBV, and FM, while a score of 3 represents the maximum extent of the clinical symptom/eye. As shown in Figure 2A, the control eye (Fig. 2A) did not display any clinical sign of uveitis. LPS treatment (Fig. 2B) induced severe miosis as evidenced by SP, DBV, and FM (Fig. 2B). The LPS group treated with ME (Fig. 2C) showed a remarkable reduction in the clinical signs (Fig. 2C). Figure 2B shows the difference in the clinical scores brought about by topical application of ME and PBS onto the eyes of LPS-treated rats. The clinical scores for the EIU rats were 2.88 ± 0.13 (SP); 3.00 ± 0.00 (DBV); and 1.5 ± 0.20 (FM) and were significantly reduced to 1.5 ± 0.29 (SP; *P* < 0.05); 1.5 ± 0.29 (DBV; *P* < 0.05); and 0.25 ± 0.14 (FM; *P* < 0.05) following treatment with ME.

**Figure 3:**
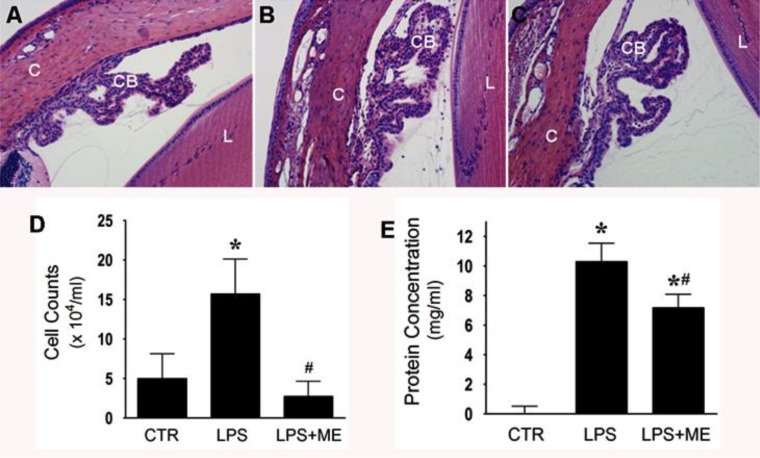
Treatment with ME reduces leukocyte infiltration and elevated protein in aqueous humor in LPS induced EIU. (A–C) Serial sections of paraformaldehyde-fixed rat eyes were stained with H&E and observed under bright field microscopy. C, cornea; CB, ciliary body; L. Lens: ×200 magnification. A representative photomicrograph is shown from each experimental group. (A) Control with PBS eye drops. (B) EIU 24 h with PBS eye drops. (C) EIU 24 hour with ME eye drops. (D, E) Leukocyte infiltration and protein concentration in aqueous humor. (D) Amelioration of LPS-induced inflammatory cell infiltration in aqueous humor by topical ME treatment. The inflammatory cells in aqueous humor were counted by the trypan blue exclusion method. (E) Reduction of total protein concentration in the aqueous humor as measured by Bradford method. *P < 0.05 versus CTR. #P < 0.05 versus LPS. ME treatment did not totally abolish the leukocyte infiltration especially protein leakage, suggesting dose adjustment or combinatorial therapy.

We examined leukocyte infiltration in rat eye sections stained with H&E or by counting the cells in the aqueous humor. As shown in the representative H&E stained sections of the eye, numerous leukocytes were observed in the aqueous humor of untreated EIU rat eyes (Fig. 3B) while in ME-treated EIU rat eyes, almost no infiltrating cells were observed (Fig. 3C). Control rat eyes showed very few infiltrated cells in the aqueous humor (Fig. 3A). Similar results were observed upon counting the cells in the extracted aqueous humor (Fig. 3D). Next we measured total protein concentration in the aqueous humor. As shown in Figure 3E an approximately 10-fold increase in protein levels was observed in the EIU rats as compared to control rats. Topical application of ME reduced the protein levels to approximately 7-fold compared to untreated EIU rats. Prevention of protein leakage into the aqueous by ME was statistically significant, but not remarkable that points toward further optimizing the concentration of the formulation.

### Effect of ME on Endotoxin-Induced Inflammatory Molecules

**Figure 4:**
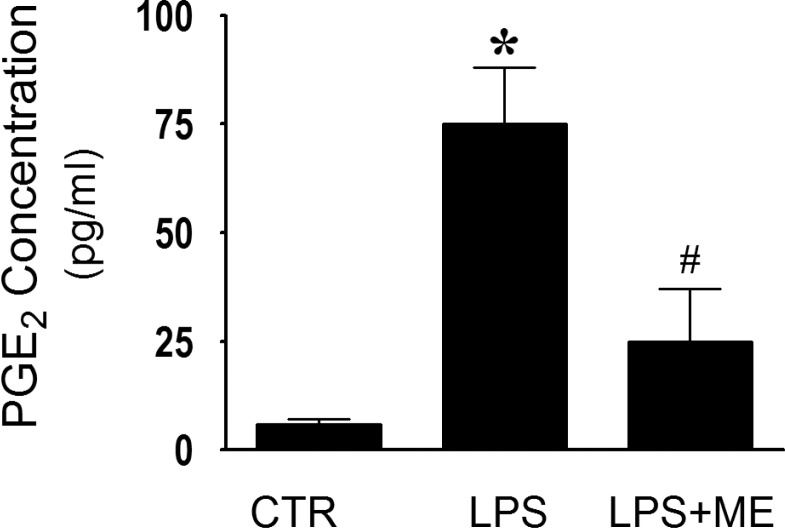
Amelioration of PGE_2_ secretion in EIU by topical application of ME. PGE_2_ levels in the aqueous humor were measured by ELISA. LPS significantly elevated the levels of PGE_2_, 6 hours after injection. Treatment with ME decreased the levels of PGE_2_ significantly. P < 0.05 versus CTR. #P < 0.05 versus LPS.

Using an ELISA assay, we observed significantly enhanced secretion of PGE_2_ (∼11-fold) in the aqueous humor of EIU rat eyes at 6 hours postinjection (Fig. 4). Treatment with ME reduced the release of PGE_2_ in the EIU rat eye by approximately 4.5-fold and brought it down to the levels that were not significantly different from the control.

**Figure 5:**
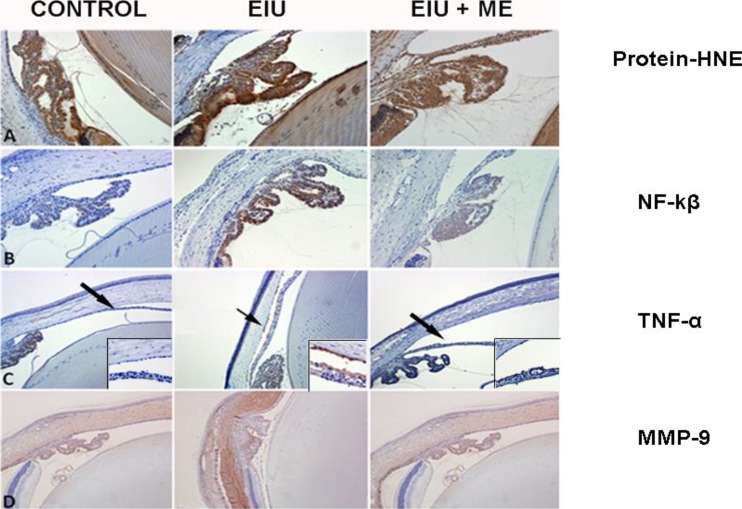
Topical application of ME ameliorated several molecular markers of inflammation in the rat eye. Immunohistochemistry of the oxidative and inflammatory markers was performed as described in the text. (A) Protein-HNE adduct formation after 24 hours of LPS challenge was prominent in epithelial cells of the ciliary body and epithelial cells of the lens (×400 magnification). (B) Immunostaining of NF-kβ after 6 hours of LPS challenge was elevated in the epithelial cells of the ciliary body (×200 magnification). Secretion of TNF-α (C) was observed in the anterior chamber (×100 magnification, inset ×400) and MMP-9 (D) was observed within the cornea and sclera (×100 magnification) after 24 hours of LPS challenge. Treatment with ME decreased the expression of all of these toxic markers.

We examined the effect of ME eye drops on EIU-induced ROS (protein-HNE adducts) and various inflammatory markers 24 hours after LPS challenge; NF-κB was evaluated 6 hours after LPS challenge since activation of this transcription factor is an early event in the inflammatory cascade. Figure 5A shows increased immunostaining of protein-HNE adducts corresponding to an increased level of ROS in the anterior segment of the eyes, including the iris-ciliary body complex. Application of the ME formulation reduced LPS-induced increases in staining for protein-HNE adducts in ocular tissues in EIU rat eyes. Immunostaining of rat eye sections with antibodies against phospho-p65 (the active subunit of NF-κB), showed increased intensity for NF-κB staining at the iris-ciliary body complex as well as at the retinal wall (Fig. 5B). The increase in NF-κB staining observed after EIU was reduced by treatment with ME. No NF-κB staining was observed in control rat eyes. Similarly, we examined the effect of ME downstream of NF-κB activation by looking at the expression of NF-κB dependent inflammatory proteins. The EIU rat eyes showed increased expression of TNF-α and MMP-9 proteins in the anterior segment of the eye (Figs. 5C, 5D) as indicated by increased staining for these antigens. Treatment with ME reduced the expression of TNF-α as well as MMP-9 proteins.

## Discussion

The results of this study demonstrate the efficacy of topically administered ME in reducing the acute inflammatory response of EIU. EIU is a commonly used model of human acute anterior uveitis. It is a self-limiting disease whose symptoms peak approximately 24 hours postinjection and spontaneously resolves within a week.^23,24^ Application of ME every 2 to 4 hours as compared to topical PBS reduces the clinical signs of acute inflammation, migration of leukocytes and leakage of protein into the aqueous humor, and expression of several inflammatory markers that occur within hours of subcutaneous injection of *Escherichia coli* LPS. Uveitis model is an acute model of inflammation and oxidative stress. Therefore, this 5X concentration of the previous study,^21^ which was a chronic model of oxidative stress, was used. Furthermore, based upon the proprietary MSM/EDTA formulation of Livionex, Inc., and our previous studies,^21^ the ratio of EDTA-MSM was maintained at a molar ratio of 1:8.

Oxidative stress–induced ROS generation is a major factor in triggering inflammation and tissue damage during the inflammatory process induced by LPS.^2^ Upon activation, polymorphonuclear leukocytes and monocyte/macrophages undergo a respiratory burst marked by a large increase in oxygen uptake and increased production and release of ROS including superoxide and hydrogen peroxide.^25,26^ Hydrogen peroxide is converted via the metal-catalyzed Fenton reaction to the damaging radical •OH. The generation of ROS is catalyzed by the redox active metal ions of copper (Cu^2+^) and iron (Fe^2+^, Fe^3+^).^27^ Oxidative damage worsens as transition metal ions catalyze free-radical reactions and injured cells release more metal ions into the extracellular space. The increased level of extracellular ions catalyzes the free-radical reactions creating more oxidative damage and widening the area of injury. High levels of iron in the body are implicated in a variety of disorders including cardiovascular diseases, neurodegeneration, and cancer.^16–18^

There is evidence that metal ions are involved in uveitis. Clinically, bleeding into the eye has long been known to exacerbate eye inflammation due to the iron in hemoglobin. Injection of hemoglobin along with LPS into rabbit eyes significantly exacerbated clinical inflammation.^28^ During EIU in rabbits, iron concentration and the total iron-binding capacity of the aqueous and vitreous humors rise.^29^ Normally, the total iron-binding capacity is well below saturation but comes close to total saturation during inflammation. Copper ions are higher in the plasma of patients with Behçet disease, an inflammatory disease usually accompanied by uveitis, implicating this metal in the disease.^30^

Chelation therapy has been proposed for treatment of many diseases linked to oxidative stress.^16–18^ Given the copious evidence linking metal-catalyzed ROS to ocular inflammation; it is surprising that research using chelation therapy for the treatment of uveitis is sparse. In the late 80s and early 90s, several experiments were done by Rao et al.,^31^ looking directly at chelation and oxidative stress in EAU in rabbits. A chelator of ferric iron (Fe^3+^), deferoxamine mesylate, resulted in a marked decrease of ocular inflammation in EAU.^31^ Deferoxamine also reduced the products of lipid peroxidation.^32^ EDTA was used by Mahlberg et al.^33^ to reduce inflammation in experimental autoimmune uveitis (EAU). They observed a decrease in phospholipase A2 and myeloperoxidase activity, inflammatory mediators derived from the arachadonic acid pathway.

The ME eye drop formulation combines EDTA with MSM. Pharmacokinetics studies in rat eyes were performed using radiolabeled EDTA in the absence and presence of MSM.^20^ EDTA is generally added to biologic solvents and topical eye drops to suppress the interference of nonprotein bound metal ions. EDTA is readily excreted by the kidneys and has been approved by the FDA for the treatment of lead poisoning and other metal toxicities when used intravenously. It is also used as an antioxidant in foods, as a chelating agent in many pharmaceuticals and cosmeceuticals. Alone, however, EDTA does not penetrate the cornea. MSM is a widely used dietary supplement in the nutraceutical market and has no known toxicity. Being amphipathic, MSM possesses unique solubilizing properties and facilitates the entry of EDTA into cells. Our earlier studies demonstrated that MSM is able to deliver [14C]-tagged sodium EDTA into the cornea, aqueous, lens, vitreous and retina-choroid tissues within minutes after topical administration to the eye.^20^ EDTA levels were not determined in the aqueous or the serum in the present experiments. However, based upon the previous studies in which we measured the EDTA in the ocular tissues when 2.6% EDTA was applied, the aqueous humor EDTA calculated concentration in the present study would be around 20 μM. If all the EDTA applied enters into the blood through the aqueous/eye, its calculated concentration in the serum would then be 23 μM. This concentration is at least 60-fold lower than the calculated blood EDTA concentration of ∼1.5 mM in the clinical studies where 3-g EDTA infusions were given with no apparent adverse effects. Furthermore, the biologic half-life of EDTA is ∼1 hour and would have a fast clearance that is evident from our previous studies.^21^ It is safe to assume that potential adverse effects would be minimal. However, conducting a systematic study to unequivocally demonstrate the safety of the doses used is warranted.

The anti-inflammatory effect of topically applied ME was observed on a number of clinicopathologic measures of inflammation (Fig. 2). The observed amelioration of clinical symptoms by ME correlated with reduced signs of breakdown of the blood–ocular barrier. Inflammation during EIU is characterized by a breakdown of the blood–ocular barrier resulting in an increase of total protein content in the aqueous humor, and cellular infiltration of leukocytes into the anterior chamber of the eye.^34^ ME ameliorated these aspects of EIU induced ocular inflammation, which is evident from the decreased leukocyte infiltration and protein concentrations in the aqueous humor of rat eyes (Fig. 3). Elevated protein concentration in the aqueous humor is partially due to increased expression of various inflammatory markers by invading leukocytes. These proteins include cytokines, chemokines and PGE_2_.^35^ Aqueous humor analysis showed the presence of PGE_2_ at 6 hours after LPS injection (Fig. 4). PGE_2_ expression is thought to be part of the cascade that breaks down the blood–ocular barrier, since earlier studies have paralleled the rise in protein in the aqueous humor with the rise of PGE_2_ expression.^8^ MMP-9 is also likely to be a part of this process since it cleaves type IV collagen in basement membranes and allows the extravasion of leukocytes from the circulatory system into the aqueous humor during uveitis.^10^ We found that treatment with ME reduced PGE_2_ as measured by ELISA (Fig. 4) and qualitatively reduced immunostaining for MMP-9 (Fig. 5E). Lower levels of these proteins, reduced overall protein levels ,and decreased numbers of infiltrating leukocytes all imply that ME treatment helps to maintain the blood-ocular barrier in EIU. However, protein levels decrease was not as pronounced as the infiltrating cells decrease (Fig. 3). It is possible that regional vascular function is still compromised as seen in uveitis.^1,2^ Increasing the concentrations of ME or including amino guanidine is suggestive. Corticosteroids may still be the mainstay of therapy; however, they are associated with well-established side effects. This regimen with the chelator has the potential to be a better safety and efficacious treatment.

Because the visual loss in humans with uveitis occurs mainly from retinal tissue damage, it is hypothesized that such damage results from free radical induced peroxidation of retinal cell membranes. Lipid peroxidation has been shown to occur in EAU by Ishimoto et al.^36^ They measured elevated conjugated dienes and keto-dienes, products of lipid peroxidation. Lipid peroxidation products were also found to be high in the blood of patients with Behçet disease.^37^ Byproducts of lipid peroxidation have been shown to be chemoattractant for leukocytes in EAU.^38^ Since leukocytes are a major source of ROS that leads to lipid peroxidation, this sets up a positive feedback loop that amplifies inflammation. During lipid peroxidation, several lipid-derived aldehydes are formed. One of the most studied and most toxic of these is HNE.^4^ High levels of HNE are associated with induction of proapoptotic caspase enzymes, DNA laddering, release of cytochrome C from mitochondria and cell death.^39,40^ Recent reports support a role for this aldehyde in inflammation.^41^ We have extended the finding of lipid peroxidation in inflammation to the presence of HNE in the ciliary body and lens in EIU rat eyes. Treatment with ME reduced the amount of these protein-HNE adducts.

Calcium ions serve many roles in the pathway leading to inflammation and apoptosis. Calcium has been shown to be essential for the activation of the redox-activated transcription factor NF-κB by hydrogen peroxide.^5^ NF-κB regulates the expression of a variety of genes essential for cellular immune response such as inflammation, growth, development and apoptotic processes. NF-κB is a critical mediator of LPS-induced inflammation.^7^ LPS activates NF-κB in the mouse lens^42^ and there are several reports showing elevation of NF-κB in various ocular tissues of animals with uveitis.^43^ Furthermore, inhibitors of NF-κB have been shown to prevent uveitis.^11,22,44^ In addition to chelating iron and reducing the amount of ROS, chelating Ca^2+^ may be an additional mechanism by which ME effects reductions in the amount of NF-κB and cytokine induction in EIU.

TNF-α may be the most important cytokine induced by NF-κB during uveitis.^45^ TNF-α is a pleiotropic cytokine produced by activated macrophages and monocytes during immune response to various infectious agents or other oxidant stimuli.^9^ It is one of the most abundant and toxic cytokines secreted after LPS stimulation. Several studies have documented that LPS increases TNF-α levels in the aqueous humor during uveitis.^45–47^ Indeed, the varying susceptibility of different rat strains to EIU has been positively correlated to TNF-α levels in the retina.^48^ Recent studies suggest that administering anti–TNF-α chimeric monoclonal antibodies (infliximab) to patients with acute uveitis and Behçet disease ameliorates ocular inflammation.^49,50^ TNF-α is an inducer of MMP-9 that contributes to breakdown of basement membrane in vascular epithelium as discussed previously.^10^ TNF-α has been detected in eyes of patients with Behçet disease^50^ and has been associated with a recurrent pattern of uveitis.^35^ In addition to being induced by NF-κB, TNF-α is one of the most potent physiologic inducers of NF-κB. Dudek et al.,^51^ have shown that TNF-α activates NF-κB in human lens epithelial cells. This constitutes another positive feedback loop that contributes to the amplification of inflammation.

In summary, this study of localized chelation of transition metal and calcium ions offers a new therapeutic approach to treat inflammation. Direct application of EDTA permits higher concentration to the inflamed region without disturbing important functions of transition metal and calcium ions elsewhere. An important part of being able to deliver effective anti-inflammatory concentrations is the use of the membrane permeation enhancer, MSM, which allows passage of EDTA through epithelial barriers.^20^ Our results indicate that ME prevents endotoxin-induced infiltration of leukocytes and protein into the aqueous humor and prevents expression of several inflammatory markers in eye tissues and suggest that ME formulation is potentially therapeutic in ocular inflammation, especially uveitis.

## Supplementary Material

Supplement 1
