# Supplementary material for: Amelioration of Endotoxin-Induced Inflammatory Toxic Response by a Metal Chelator in Rat Eyes
Source: Invest Ophthalmol Vis Sci. 2018 Jan;59(1):31–8. doi: 10.1167/iovs.17-22172 (PMC5754197; doi:10.1167/iovs.17-22172)
Supplement: Supplement 1 [file iovs-58-14-35_s01.pdf]

Supplemental Material:

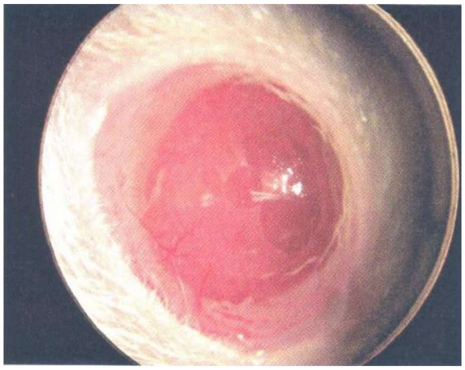

MSM 2.7% topically applied to the eyes of EIU rats did not have a preventative effect on the development of Uveitis.
